# The Role of Estrogen in Anxiety-Like Behavior and Memory of Middle-Aged Female Rats

**DOI:** 10.3389/fendo.2020.570560

**Published:** 2020-10-07

**Authors:** Emese Renczés, Veronika Borbélyová, Manuel Steinhardt, Tim Höpfner, Thomas Stehle, Daniela Ostatníková, Peter Celec

**Affiliations:** ^1^Institute of Molecular Biomedicine, Faculty of Medicine, Comenius University in Bratislava, Bratislava, Slovakia; ^2^Institute of Physiology, Faculty of Medicine, Comenius University in Bratislava, Bratislava, Slovakia; ^3^Institute of Pathophysiology, Faculty of Medicine, Comenius University in Bratislava, Bratislava, Slovakia; ^4^Department of Molecular Biology, Faculty of Natural Sciences, Comenius University in Bratislava, Bratislava, Slovakia

**Keywords:** exploration, gonadectomy, masculinization, mood disorders, senescence

## Abstract

Aging in women is associated with low estrogen, but also with cognitive decline and affective disorders. Whether low estrogen is causally responsible for these behavioral symptoms is not clear. Thus, we aimed to examine the role of estradiol in anxiety-like behavior and memory in rats at middle age. Twelve-month old female rats underwent ovariectomy (OVX) or were treated with 1 mg/kg of letrozole—an aromatase inhibitor. In half of the OVX females, 10 μg/kg of 17β-estradiol was supplemented daily for 4 weeks. Vehicle-treated sham-operated and OVX females served as controls. For behavioral assessment open field, elevated plus maze and novel object recognition tests were performed. Interaction between ovarian condition and additional treatment had the main effect on anxiety-like behavior of rats in the open field test. In comparison to control females, OVX females entered less frequently into the center zone of the open field (*p* < 0.01) and showed lower novel object discrimination (*p* = 0.05). However, estradiol-supplemented OVX rats had higher number of center-zone entries (*p* < 0.01), spent more time in the center zone (*p* < 0.05), and showed lower thigmotaxis (*p* < 0.01) when compared to OVX group. None of the hormonal manipulations affected anxiety-like behavior in the elevated plus maze test significantly, but a mild effect of interaction between ovarian condition and treatment was shown (*p* = 0.05). In conclusion, ovariectomy had slight negative effect on open-field ambulation and short-term recognition memory in middle-aged rats. In addition, a test-specific anxiolytic effect of estradiol supplementation was found. In contrast, letrozole treatment neither affected anxiety-like behavior nor memory.

## Introduction

Post-menopause is a life period in women accompanied by reproductive senescence, including decline in ovarian sex hormones (1). Besides physical changes (2), there are many mental and psychological disorders related to post-menopausal syndrome, such as depression, anxiety or dementia (3–6). The underlying mechanism of age-related cognitive decline and anxiety is unclear, but the role of estrogen loss is considered (7–10). However, most of the post-menopausal symptoms may result from the aging process as well as from loss in ovarian endocrine function (11). To analyze the causal effect of menopause-related decline in sex hormone production, further experimental studies are needed.

In animal experiments, the bilateral removal of ovaries—ovariectomy—is the most used tool to mirror the post-menopausal state of women (12). It has been shown that ovariectomy causes impaired learning and memory indicated by longer latency time to find a platform and less time spent in the target zone in the Morris water maze, and by lower recognition index in the novel object recognition and object placement tasks (13–16). Similarly, there are numerous data showing anxiogenic effect of ovariectomy in several tests, such as open field, elevated plus maze or light-dark box (16–20). On the other hand, estradiol replacement in the surgical model of menopause may improve cognitive functions and decrease anxiety-like behavior in rodents (14, 21–31).

Inhibition of aromatase leads to decreased estrogen production as well, while it increases gonadotropin releasing hormone or luteinizing hormone, and causes hyperandrogenism (32–34). Animal studies using pharmacological aromatase inhibitors - e.g., letrozole, and aromatase knock-out mice have suggested that aromatase has a role in cognitive functions (35). Neurobehavioral studies have found that aromatase inhibition may result in decreased spine and synaptic density in forebrain, impaired spatial memory, recognition memory, and contextual fear memory (36, 37). On the contrary, letrozole treatment in middle-aged females may increase neurogenesis in the hippocampus (38). Although letrozole might be efficient for treatment of infertility at reproductive age, e.g., in women with polycystic ovary syndrome (39), its most common clinical application is in postmenopausal women suffering from breast cancer (40).

Natural menopause in women occurs typically at midlife, i.e., 50 years (41). Therefore, studies performed in middle-aged rats are valuable and important for understanding the causal relationship between estrogen deficiency and psychological features of post-menopausal syndrome. Although there are published studies on the effect of ovariectomy (29, 30, 42–44) and estradiol treatment (21–23, 25, 29, 31) on memory and anxiety-like behavior in middle-aged (~12-month old) and old rodents (>18-month old), studies on such behavioral effects of letrozole treatment are rare (45).

In this study, we consider that ovariectomy is accompanied by decreased production of ovarian sex hormones including androgens, while inhibited aromatization of testosterone may lead to its excess. Thus, our main goal was to compare and explore the causal effect of both, surgical and pharmacological inhibition of estrogen production on anxiety-like behavior and memory in middle-aged rats. We hypothesized that if estrogen deficiency is the main cause of cognitive dysfunction and anxiety in middle-aged females, both ovariectomy and letrozole-treatment will impair memory and induce higher anxiety-like behavior in comparison to controls. In addition, we aimed to examine whether estradiol supplementation may improve memory and attenuate anxiety in middle-aged ovariectomized female rats.

## Methods

### Animals

Female Wistar rats (*n* = 42, 12-month old, weighing 362 ± 52 g) were used in the experiment. Twelve-weeks old animals were purchased from Velaz (Prague, Czech Republic), and maintained under standard conditions (temperature 25 ± 2°C and humidity 55 ± 10%) with a 12:12 light-dark cycle, and housed in groups, 4-5 per cage (w:38 × l:60 × h:20 cm). Except the duration of behavioral testing, animals had *ad libitum* access to food and water. The experiment was approved by the local Ethics committee and performed according to the Slovak legislation.

### Ovariectomy

Twelve-months old female rats underwent either ovariectomy (OVX, *n* = 21) or sham surgery (F, *n* = 21). The procedures were performed under general anesthesia using intraperitoneal injection of ketamine (100 mg/kg) and xylazine (10 mg/kg). A single ventral transverse incision of 1–1.5 cm was made at the middle abdominal region. The uterine horns and vessels were ligated on both sides, the ovaries were cut, and the remaining tissue was placed back into the abdominal cavity. The muscle and skin layers were sutured with absorbable silk (size 4-0).

### Hormonal Treatment

Five weeks after surgery, females were randomly divided into several treatment groups. For 4 weeks, sham-operated females were treated with an aromatase inhibitor, letrozole, in a dose of 1 mg/kg (Sigma-Aldrich, Darmstadt, Germany; F+LET, *n* = 11), and OVX females with 17β-estradiol in a dose of 10 μg/kg (Sigma-Aldrich, Darmstadt, Germany; OVX+E, *n* = 11). Vehicle-treated F and OVX groups received olive oil in a volume of 1 ml/kg (Olivae Oleum Raffinatum, Galvex, Banská Bystrica, Slovak Republic). The treatments were administered once daily, between 3 and 5 p.m., by subcutaneous injection.

### Behavioral Testing

During the last week of treatment, the animals were tested for locomotor activity, anxiety-like behavior and memory using a battery of behavioral tests. The behavioral phenotyping of rats was carried out in three consecutive days (one test per day), between 8 and 12 a.m., in the fixed order from the least to the most stressful, i.e., open field, novel object recognition, and elevated plus maze test. Each test was recorded using a camcorder placed above the apparatus in the middle of the testing room. All observed parameters were analyzed using the image and video processing system EthoVision XT 10.0 (Noldus Information Technology, Wageningen, Netherlands).

#### Open Field Test

Open field test was performed to assess locomotor activity and anxiety-like behavior of animals. A square shaped (100 × 100 cm) apparatus was used, virtually divided into a center (40 × 40 cm) and border zone, which was slightly illuminated with white light (25 lx). Animals were placed individually into the center zone, and were allowed to freely explore the arena for 5 min. Time spent in and number of entries into the center zone, as well as distance to the wall indicating thigmotaxis was monitored as indexes of anxiety-like behavior.

#### Novel Object Recognition Test

The novel object recognition test was conducted in the familiar apparatus and under the same condition as the open field test. During the training phase, the animals were exposed to two identical objects (two green plastic or two transparent glass bottles) for 5 min. Total time of interactions with any of the objects was analyzed to assess explorative behavior. One hour later, one of the two objects was swapped for a novel object with a different shape, material and color as the familiar one, and the animals were returned into the arena for another 5 min. To avoid any preference of side or features, the objects (green plastic/transparent glass bottle) were randomly selected as familiar or novel, as well as the position of the novel object in left or right side was systematically altered between the trials. To assess memory, novel-object discrimination was calculated as following: interaction with novel object/(interaction with novel + familiar object)^*^100. The animals were excluded from analysis, if the object exploration during the training and/or during the testing was below 5% (<15 s from 5 min).

#### Elevated Plus Maze Test

A plus-shape apparatus elevated to a height of 60 cm above the floor was used to assess anxiety-like behavior. The arena consisted of two opposite open with 100–110 lx illumination and two opposite closed arms with 3–5 lx illumination, extending from central platform. Animals were placed onto the central platform facing to an open arm, and allowed to explore the maze for 5 min. The open-arm preference was evaluated as the number of entries into [open-arm entries/total entries] and time spent in the open arms [time on open arms/(time in open + closed arms)] relative to entries and time in any of the arms.

### Uterus Weight and Testosterone Concentration

To verify the effect of surgery and hormonal replacement, uterus weight and testosterone concentration were assessed. At sacrifice of the animals, blood was taken from abdominal aorta and the uterus was dissected. Uterus weight was measured on an analytic scale, and adjusted to body weight (46). Blood samples were centrifuged at 2,400g for 5 min, and plasma samples were stored at −20°C until analysis. The concentration of testosterone in plasma was measured using a commercial ELISA kit (DRG Diagnostic, Marburg, Germany) with 0.029 nmol/L analytical sensitivity, and <5% inter- and intra-assay coefficients of variations.

### Statistical Analysis

For statistical analysis GraphPad Prism version 6.00 for Windows (GraphPad Software, La Jolla, CA, USA) was used. Data were analyzed using two-way ANOVA – one factor being ovarian condition (sham, OVX) and the other factor being additional treatment (control, letrozole in sham groups or estradiol in OVX group). Bonferroni multiple comparison *post hoc* test was used to compare mean of each group with means of every other groups. *P*-values have been adjusted to account for multiple comparison bias. Differences were considered statistically significant when *p* < 0.05. Data are shown as mean plus standard deviation (SD).

## Results

**Figure 1:**
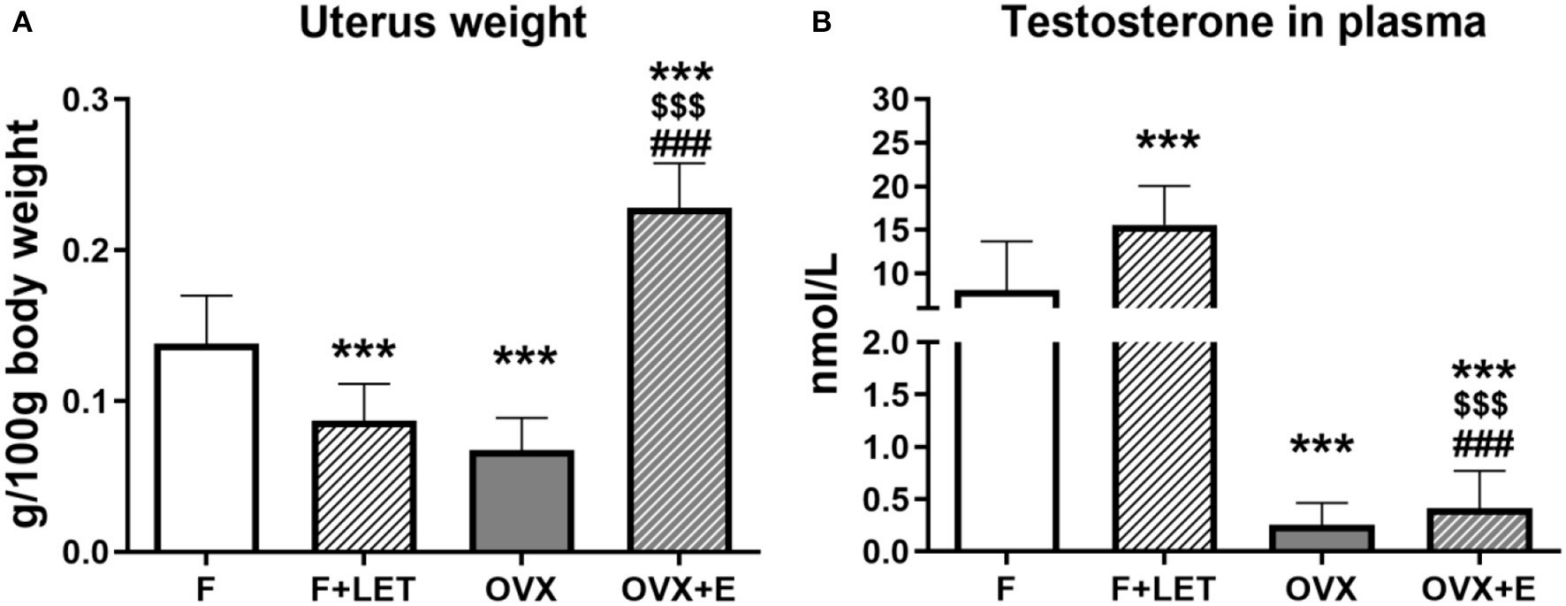
Uterus weight adjusted to body weight **(A)** and testosterone concentration in plasma **(B)**. Data are presented as mean + SD. ^***^p < 0.001 in comparison to F group, ^$$$^*p* < 0.001 in comparison to F + LET group, ^###^*p* < 0.001 in comparison to OVX group; *n* = 9–11/group; F, females; F + LET, letrozole-treated females; OVX, ovariectomized females; OVX + E, estradiol-treated ovariectomized females.

To confirm the endocrine changes induced by ovariectomy, estradiol supplementation and letrozole treatment, uterus weight and plasma testosterone were measured. There was a main effect of ovarian condition [F_(1, 37)_ = 17.0, *p* < 0.001], a main effect of treatment [F_(1, 37)_ = 40.7, *p* < 0.001] and a significant interaction between these two factors [F_(1, 37)_ = 153, *p* < 0.001] on uterus weight (Figure 1A). In comparison to the F group, the weight of the uterus was significantly lower in OVX (*p* < 0.001) as well as in F + LET group (*p* < 0.001). OVX + E females had a higher uterus weight when compared to OVX group (*p* < 0.001), F group (*p* < 0.001) or F + LET group (*p* < 0.001).

A main effect of ovarian condition [F_(1, 36)_ = 98.1, *p* < 0.001], a main effect of treatment [F_(1, 36)_ = 11.1, *p* < 0.01], and a significant interaction between ovarian and treatment condition [F_(1, 36)_ = 10.2, *p* < 0.01] was observed also on concentration of testosterone (Figure 1B). Both, the OVX (*p* < 0.001) and OVX + E groups (*p* < 0.001) had lower, while F + LET group had higher (*p* < 0.001) concentration of testosterone in comparison to control F group. The F + LET group had higher testosterone than OVX (*p* < 0.001) and OVX + E group (*p* < 0.001). OVX + E did not differ from OVX in testosterone concentration (*p* > 0.99).

**Figure 2:**
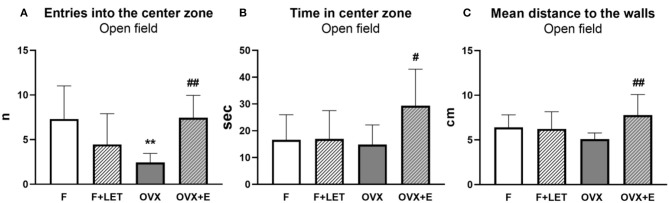
Anxiety-like behavior assessed in the open field test by number of entries into the center zone **(A)**, time spent in the center zone **(B)**, and mean distance to the walls **(C)**. Data are presented as mean + SD. **p < 0.01 in comparison to F group, ^##^*p* < 0.01 and ^#^*p* < 0.05 in comparison to OVX group; *n* = 9–11/group; F, females; F + LET, letrozole-treated females; OVX, ovariectomized females; OVX + E, estradiol-treated ovariectomized females.

A significant interaction between ovarian condition and treatment was found [F_(1, 37)_ = 18.5, *p* < 0.001], but neither ovarian condition [F_1, 37_=1.03, *p* = 0.32] nor treatment [F_(1, 37)_ = 1.41, *p* = 0.24] had a main effect on the number of entries into the center zone in the open field test (Figure 2A). OVX group entered less frequently into the center zone than F group (*p* < 0.01). The number of entries was higher in OVX + E when compared to OVX (*p* < 0.01). F + LET (*p* = 0.19) and OVX + E (*p* > 0.99) groups were comparable to F. In addition, the F + LET group neither differed from OVX group (*p* = 0.80) in center-zone entries.

On anxiety-like behavior assessed by time spent in the center zone in the open field test (Figure 2B), a significant main effect of treatment [F_(1, 37)_ = 5.02, *p* < 0.05], and a significant interaction between ovarian condition and treatment [F_(1, 37)_ = 4.60, *p* < 0.05] was found, while no effect of ovarian condition alone was observed [F_(1, 37)_ = 2.60, *p* = 0.12]. OVX + E group spent more time in the center zone in comparison to the OVX group (*p* < 0.05), F group (*p* = 0.05) and F + LET group (*p* = 0.06). Neither OVX (*p* > 0.99) nor F+LET (*p* > 0.99) groups differ from F. In addition, the OVX group was comparable to F + LET group (*p* > 0.99).

There was a main effect of treatment [F_(1, 37)_ = 5.37, *p* < 0.05], and a significant interaction between ovarian condition and treatment [F_(1, 37)_ = 7.02, *p* < 0.05], but no effect of ovarian condition [F_(1, 37)_ = 0.04, *p* = 0.84] on thigmotaxis assessed by distance to the walls during the open-field exploration (Figure 2C). Animals in OVX + E group explored the arena farther from the walls in comparison to the animals in OVX group (*p* < 0.01). No other differences were found between the groups (OVX vs. F: *p* = 0.62; F + LET vs. F: *p* > 0.99; OVX + E vs. F: *p* = 0.47; F + LET vs. OVX: *p* = 0.90; OVX + E vs. F + LET: *p* = 0.26).

**Figure 3:**
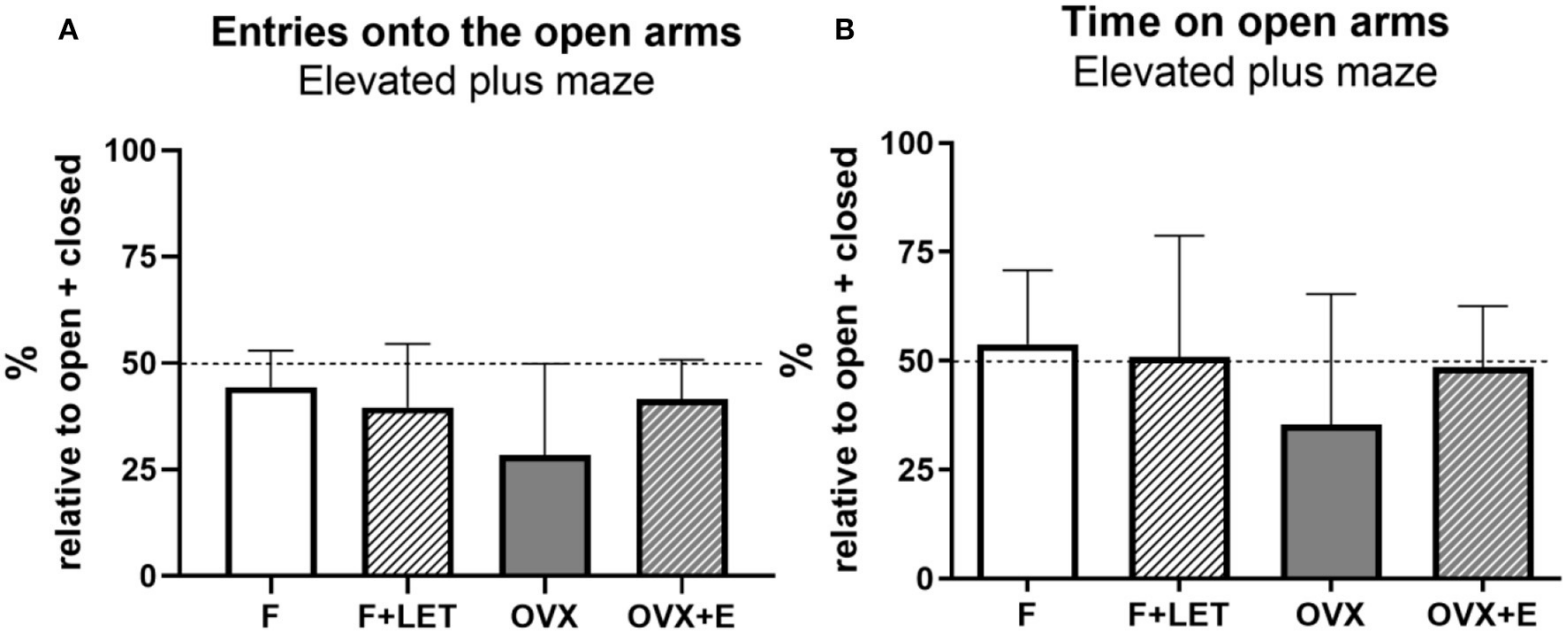
Anxiety-like behavior assessed in the elevated plus maze test by number of entries onto the open arms **(A)** and time spent on the open arms **(B)**. These parameters are expressed as percentage of total entries made into or time spent in the closed + open arms, respectively. Dashed line indicates equal frequency and time exploring open and closed arms. Data are presented as mean + SD. *n* = 10–11/group; F, females; F + LET, letrozole-treated females; OVX, ovariectomized females; OVX + E, estradiol-treated ovariectomized females.

In the elevated plus maze test, no significant main effect of ovarian condition [F_(1, 37)_ = 2.41, *p* = 0.13] or treatment [F_(1, 37)_ = 0.87, *p* = 0.36], and a statistically non-significant interaction between ovarian condition and treatment [F_(1, 37)_ = 3.94, *p* = 0.05] was found on the number of entries onto the open arms (Figure 3A). Similarly, neither ovarian condition [F_(1, 38)_ = 2.13, *p* = 0.15], nor treatment [F_(1, 38)_ = 0.52, *p* = 0.47], nor interaction between these two factors [F_(1, 38)_ = 1.27, *p* = 0.27] affected anxiety-like behavior assessed by time spent on the open arms (Figure 3B).

**Figure 4:**
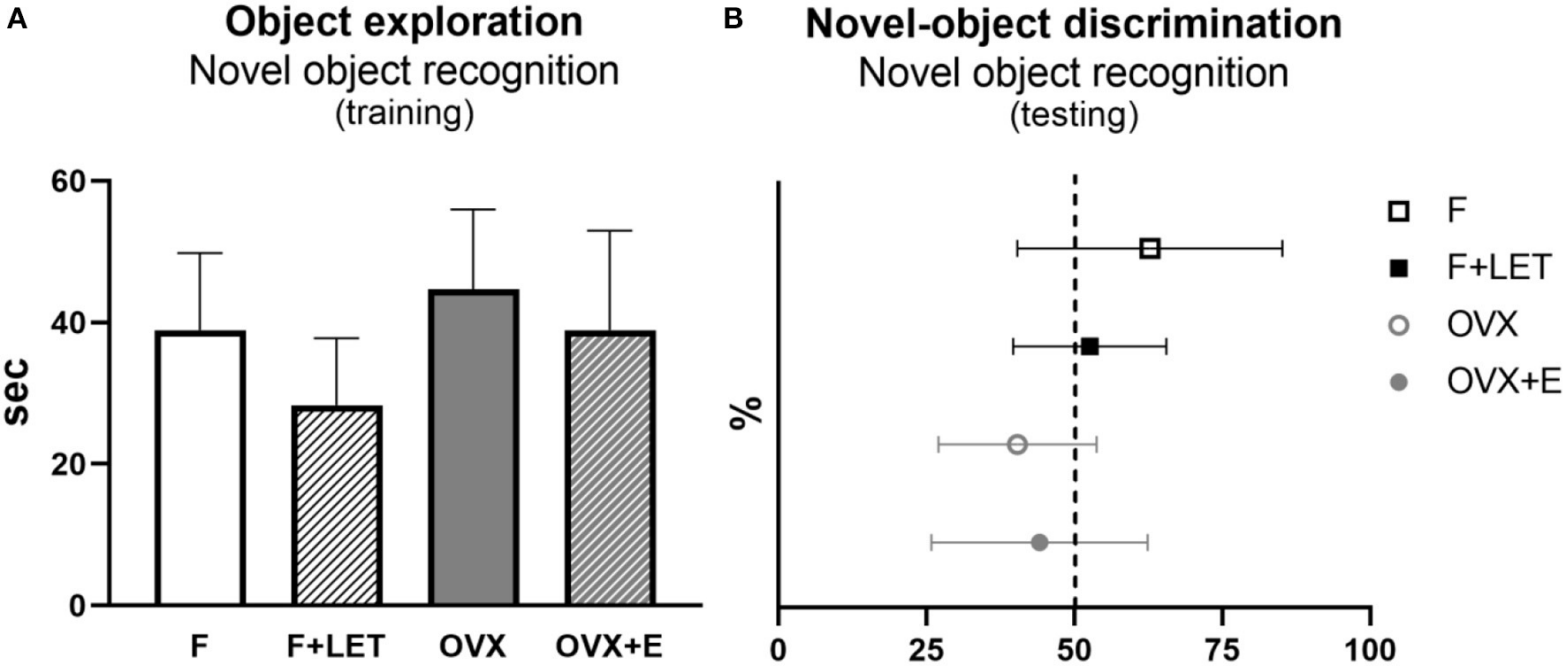
Exploratory behavior assessed by total time spent exploring the objects during the training period **(A)** and recognition memory assessed by novel-object discrimination during the testing period in the novel object recognition test **(B)**. Novel-object discrimination is expressed as percentage of total time spent exploring the novel and familiar objects. Dashed line indicates equal time spent exploring novel and familiar objects. Data are presented as mean + SD. *n* = 6–11/group; F, females; F + LET, letrozole-treated females; OVX, ovariectomized females; OVX + E, estradiol-treated ovariectomized females.

In the training phase of the novel object recognition test (Figure 4A), no effect of ovarian condition [F_(1, 29)_ = 2.69, *p* = 0.11] or treatment [F_(1, 29)_ = 2.67, *p* = 0.11], and neither a significant interaction between these two factors [F_(1, 29)_ = 0.07, *p* = 0.80] was shown, while some animals had to been excluded from analysis (*n* = 5 in F + LET group and *n* = 4 in OVX group) due to a low object-exploration time. To assess short-term recognition memory, we analyzed novel-object discrimination in the testing phase of the novel object recognition test (Figure 4B). Animals exploring the objects for <15 s during the training and/or in testing phase have been excluded from analysis (*n* = 1 in F, *n* = 5 in F + LET, *n* = 4 OVX, *n* = 1 OVX + E). There was a significant main effect of ovarian condition [F_(1, 27)_ = 5.40, *p* < 0.05], but no significant effect of treatment [F_(1, 27)_ = 0.24, *p* = 0.63] or an interaction between ovarian condition and treatment [F_1, 27_ = 1.09, *p* = 0.30] on novel object discrimination. Based on the *post-hoc* test, the OVX group (*p* = 0.05) exhibited lower discrimination of the novel object when compared to F group.

## Discussion

In this experiment, ovariectomy in middle-aged females had a slight effect on open-field ambulation and on recognition memory. On the contrary, letrozole treatment had no effect either on anxiety-like behavior or memory in females. Estradiol supplementation in OVX females had test-specific anxiolytic effect in the open field test, but no effect on memory in the novel object recognition task.

There is some evidence indicating that ovariectomy in young-adult females has anxiogenic effect (16–20). However, there is little data on the effect of ovariectomy on anxiety in middle-aged rats (44). Extensive research has been dedicated to examine anxiolytic effect of estradiol in young-adult OVX females (27, 47–52), while only few studies are available on the effect of estradiol on anxiety-like behavior in aged OVX (21) or gonadally intact females (53). Recently, lower anxiety-like behavior was found in 16–18-months old OVX females when compared to the intact counterparts 14 weeks after ovariectomy in elevated plus maze and light-dark box test (44). On the contrary, we found only a mild effect of ovariectomy on anxiety-like behavior in 12-month-old females in open field or in elevated plus maze test assessed 8 weeks after surgery. The differences might be caused by different age and/or length of ovarian hormone deficiency (54, 55). In our experiment, lower number of entries into the center zone was observed in OVX females when compared to the sham-operated F group, while these two groups were comparable in time spent in the center zone or in distance to walls during the open-field ambulation. However, OVX + E group entered more frequently and also spent more time in the center zone, as well as showed lower thigmotaxis in comparison to OVX females. It is possible that ovariectomy decreases exploratory behavior (13) without any effect on anxiety-like behavior in the open field, while estradiol treatment in OVX females may rescue this impairment in exploration and may decrease anxiety-like behavior as well (21). These results indicate differences in molecular mechanisms of endogenous and exogenous estrogens (28). On the other hand, estradiol treatment did not affect anxiety-like behavior of OVX rats in the elevated plus maze test. The time period between ovariectomy and hormone replacement (21), as well as the length of estradiol treatment (56) are crucial factors modulating anxiolytic effect of estradiol. Hormonal changes may down- or up-regulate the expression of specific estrogen receptor subtypes, which mediate either anxiogenic or anxiolytic effect (57). Thus, the age- and ovariectomy-related changes in expression pattern of estrogen receptors, and the sensitive time-window for estrogen action in different brain regions underlying anxiety should be examined to better understand the test-specific effect of estradiol in aging OVX rats (58).

A large number of studies have shown that ovariectomy may induce cognitive dysfunction in young (13–16) and also in middle-aged rats (29, 30, 43), while estrogens may improve learning and memory performance following ovariectomy in both, young (15, 24, 59–61) and middle-aged females (21–23, 25, 29, 31). However, OVX females in our experiment exhibited mild memory impairment only, and the estradiol-treated group did not outperform vehicle-treated OVX rats. Discrepancies between our and previously published results may arise again from differences in length of exposure to endogenous ovarian hormones (30), in delay period between ovariectomy and estradiol supplementation (21, 62–64), and in dose and frequency of treatment (65). Furthermore, the effect of age should be considered. Age-dependent changes have been shown in estrogen signaling in memory-related brain regions (66, 67), indicating an age-dependent effect of ovariectomy and estrogen supplementation on cognitive functions in rats (68–70). On the contrary, there is some evidence indicating that estradiol treatment in young adult (5-month old) and old (24-month old) ovariectomized mice may improve memory in object recognition task in the same manner (71). The type of memory assessed by different tasks should also be taken into account. Most of the published experiments in middle-aged rats use tasks for hippocampal-dependent spatial memory (29, 30, 30, 43), while in our experiment the non-spatial recognition memory was examined. Moreover, the published studies examining the effect of estradiol on cognition in females are mostly focused on long-term consequences, particularly on memory consolidation (64), but rarely on memory perception or acquisition as we did in our experiment. In contrast to spatial and fear memory tested in the Morris water maze, radial arm maze, T-maze or Pavlovian learning tasks, recognition memory assessed in the novel object recognition test does not include stress stimuli, such as shock, food deprivation or swimming (72, 73). As shown, ovariectomy in middle-aged rats may induce several alterations in hippocampal gene expression influencing neurogenesis, synaptic plasticity and immune modulation (74). However, recognition memory may involve other crucial circuits besides hippocampus, e.g., medial prefrontal cortex (75, 76). To better understand the mechanisms for impaired spatial and recognition memory, further studies are required examining the effect of ovariectomy on expression pattern of specific genes in cognition-associated brain regions in middle-aged female rats.

It should be noted that aging (77) as well as letrozole-treatment (78) and ovariectomy (79) increase luteinizing hormone (LH) production. Many age-related disorders, including the Alzheimer's disease, have been attributed to this LH excess (77). However, it has been shown that the LH rise is smaller if ovariectomy is performed at old age in comparison to the effect of ovariectomy performed in young adulthood (79). This may explain differences between our results obtained from middle-aged rats and previously published data from young-adult ovariectomized females, although LH was not assessed in our study.

Ovariectomy induces loss of ovarian hormones, including estrogens, androgens as well as progesterone (80). On the contrary, inhibition of testosterone aromatization decreases peripheral estrogen production, while the concentrations of other sex hormones, such as testosterone and progesterone, might be maintained, increased and decreased, depending on the dose of the inhibitor - e.g. letrozole (78). To distinguish between the effect of estrogen- and androgen deficiency, we examined the consequences of both ovariectomy and letrozole-treatment in middle-aged rats. While ovariectomy had some mild effect on behavior of females in the open field and novel object tests, treatment with letrozole did not affect either anxiety-like behavior or memory of female rats. Similarly, Chaiton et al. (38) did not found any effect of chronic letrozole treatment on depressive-like behavior in middle-aged female mice (38). In addition, our findings are in line also with our previous results showing that letrozole may induce anxiety in males but not in female aging rats (45). The differences between behavioral consequences of ovariectomy and letrozole treatment indicate that other ovariectomy-induced endocrine changes besides estrogen deficiency, such as altered concentration of progesterone (81), androgen (82) or gonadotropins (18) may underly the observed effect of ovariectomy. The strength of this study is the use of both, surgical and pharmacological tools to induce estrogen deficiency. More importantly, the treatments were initiated at middle-age of rats, which may be more relevant to mirror estrogen loss associated with aging than ovariectomy in adult females. The main limitation is that young-adult animals were not included in the study to investigate the age-dependent effect. Furthermore, only testosterone was assessed in plasma. Immunoassays are not applicable to measure estradiol concentration in rodents, so, it was estimated indirectly via uterus weight (46). Nevertheless, as previously shown, hippocampal and not circulating estradiol is associated with age-related memory functions in female rats (83). Last but not least, there is a need to examine age-associated alterations in estrogen and androgen signaling pathways in relevant brain regions.

## Conclusion

In this experiment, we found that ovariectomy, but not letrozole, may attenuate open-field ambulation and slightly impair recognition memory in middle-aged females. Our findings indicate different neurobehavioral consequences of surgically induced estrogen deficiency and pharmacological inhibition of estrogen production. Although we failed to show the anxiogenic effect of ovariectomy, we found that exogenous estradiol may reduce anxiety-like behavior in middle-aged ovariectomized rats, at least in the open field. On the contrary, it seems that ovariectomy may impair short-term memory, but estradiol treatment does not improve it. Thus, we conclude that endocrine changes induced by ovariectomy and aromatase inhibition affect the brain differently, and the molecular mechanisms activated by exogenous estradiol may differ from the signaling pathways of endogenous estrogens. For future experiments elucidating the role of estrogen in postmenopausal syndrome, we suggest to consider some methodological issues, such as age-related changes in the neuroendocrine system or type of manipulation used to induce estrogen deficiency.

## Data Availability Statement

The raw data supporting the conclusions of this article will be made available by the authors, without undue reservation.

## Ethics Statement

The animal study was reviewed and approved by Ethics Committee of the Institute of Molecular Biomedicine.

## Author Contributions

ER and PC drafted the manuscript. ER, VB, MS, TH, and TS performed the experiment. DO and PC corrected the manuscript. All authors contributed to the article and approved the submitted version.

## Conflict of Interest

The authors declare that the research was conducted in the absence of any commercial or financial relationships that could be construed as a potential conflict of interest.
